# Comparative Analysis of Ixekizumab Effectiveness with and Without Induction Therapy in Moderate-to-Severe Psoriasis: A Real-World Study

**DOI:** 10.3390/jcm14030833

**Published:** 2025-01-27

**Authors:** Ricardo Ruiz-Villaverde, Pedro José Ezomo-Gervilla, Jose Molina-Espinosa, Manuel Galán-Gutierrez, Enrique Herrera-Acosta, Jorge Alonso Suarez-Perez

**Affiliations:** 1 Department of Dermatology, Hospital Universitario San Cecilio, Granada, Spain, Spain Biohealth Research Institute in Granada (ibs.GRANADA), 18006 Granada, Spain; pedroj.ezomo.sspa@juntadeandalucia.es (P.J.E.-G.); jose.molina.espinosa.sspa@juntadeandalucia.es (J.M.-E.); 2 Department of Dermatology, Hospital Universitario San Reina Sofía, IMIBIC, 14004 Córdoba, Spain; manuel.galan.sspa@juntadeandalucia.es; 3 Department of Dermatology, Hospital Universitario Virgen de la Victoria, 29010 Málaga, Spain; enrique.herrera.acosta.sspa@juntadeandalucia.es (E.H.-A.); jorgea.suarez.sspa@juntadeandalucia.es (J.A.S.-P.)

**Keywords:** psoriasis, biological treatment, ixekizumab

## Abstract

**Background:** Ixekizumab, an IL-17A inhibitor, is an effective treatment for moderate-to-severe plaque psoriasis. Although clinical trials support the use of an induction phase for optimal results, real-world evidence comparing induction versus maintenance-only regimens is limited. **Objectives:** This study assessed the real-world effectiveness, safety, and drug survival of ixekizumab with and without an induction phase in patients with moderate-to-severe plaque psoriasis. **Methods:** A multicenter, observational study was conducted with 183 patients treated with ixekizumab over five years at tertiary hospitals in Andalucía, Spain. Patients were divided into two groups: an induction group (160 mg at baseline, followed by 80 mg at weeks 2, 4, 6, 8, 10, and 12, then every 4 weeks) and a non-induction group (80 mg every 4 weeks from initiation). Baseline characteristics, clinical outcomes (PASI [Psoriasis Activity Skin Index] and PGA [Physician Global Assessment] scores), and drug survival were analyzed. **Results:** The majority of patients were male (64.48% in the induction group, 58.74% in the non-induction group). No significant differences were found in age or BMI [body mass index] between groups. Baseline PASI and PGA scores were higher in the induction group, reflecting greater initial disease severity. Both regimens achieved significant clinical improvements, though the induction group demonstrated faster initial responses. Drug survival was lower in the induction group (*p* = 0.0033), potentially due to the higher baseline disease burden and severity in these patients. Comorbidities, including metabolic syndrome, cardiovascular risks, and psychiatric conditions, were prevalent, particularly in the induction group. **Conclusions:** Ixekizumab is effective for moderate-to-severe plaque psoriasis, with induction therapy yielding faster responses. However, lower drug survival in the induction group highlights the influence of initial disease severity on long-term outcomes. Real-world findings support the flexibility of ixekizumab across diverse patient populations, though further research is warranted.

## 1. Introduction

Psoriasis is a chronic, immune-mediated, inflammatory disease that affects approximately 2–3% of the global population, with significant geographic variability in prevalence and severity [1,2]. Plaque psoriasis, the most prevalent clinical form, accounts for 85–90% of cases and is associated with substantial impairments in quality of life, stemming from physical disfigurement, pruritus, and psychological comorbidities, including depression and anxiety [3,4]. Beyond cutaneous involvement, up to 30% of individuals with psoriasis develop psoriatic arthritis, a chronic inflammatory arthropathy characterized by progressive joint damage and functional disability [5,6]. Additionally, moderate-to-severe forms of psoriasis are frequently linked with systemic comorbidities, such as metabolic syndrome, cardiovascular diseases, and nonalcoholic steatohepatitis, further underscoring its systemic nature and public health importance [7,8].

Current therapeutic strategies for psoriasis aim to achieve sustained skin clearance. Moderate-to-severe psoriasis, often defined by a Psoriasis Area and Severity Index (PASI) >10, body surface area involvement >10%, or a Dermatology Life Quality Index (DLQI) >10, generally requires systemic treatments [7,8]. These include conventional immunosuppressants, phototherapy, and, more recently, biologic agents that target specific pathways in the inflammatory cascade [7].

Among the biologic therapies, ixekizumab has emerged as a highly effective treatment option for moderate-to-severe plaque psoriasis. Ixekizumab is a recombinant humanized IgG4 monoclonal antibody that selectively binds to interleukin-17A (IL-17A), a proinflammatory cytokine pivotal in the pathogenesis of psoriasis [9]. By inhibiting IL-17A, ixekizumab disrupts the inflammatory feedback loop responsible for keratinocyte hyperproliferation and immune cell infiltration, leading to rapid and sustained improvements in skin symptoms. Clinical trials, including the UNCOVER-1, UNCOVER-2, and UNCOVER-3 studies, have demonstrated the efficacy and safety of ixekizumab in achieving PASI-75, PASI-90, and PASI-100 responses in a significant proportion of patients at 12 weeks, with these benefits maintained during long-term follow-up. Furthermore, ixekizumab has shown superior efficacy compared to other biologics, including etanercept and ustekinumab, in head-to-head studies [10,11]. Overall, the safety profile of ixekizumab is within expectations given its mechanism of action and is very similar to that of etanercept, the active comparator in the studies presented. The rate of adverse events during the initial 12 weeks of treatment was very similar to etanercept (44%, 57.5%, 57.8%, and 54% in placebo, ixekizumab 80 mg every 4 weeks, ixekizumab 80 mg every other week, and etanercept, respectively). The most common adverse events in the initial 12 weeks of treatment with ixekizumab were nasopharyngitis, upper respiratory tract infections, injection site reactions, and headache, all reported at higher rates than the placebo [10,11]. Treatment is contraindicated in subjects with severe acute infections such as active tuberculosis [12].

The safety profile was similar during the maintenance phase, although the event rate was lower. The incidence of hypersensitivity reactions during the induction period, excluding injection site reactions, was 3.4% in ixekizumab vs. 1.9% in the placebo.

New cases and reactivations of inflammatory bowel disease have been reported, so caution and close monitoring are recommended in these patients. Likewise, the possible risk of developing malignancies and major cardiovascular events will be closely monitored during the post-marketing period.

Despite robust evidence from randomized controlled trials, real-world data on ixekizumab’s effectiveness and safety remain limited. Real-world studies are crucial to understanding treatment outcomes in diverse patient populations, including those with comorbidities or previous treatment failures, who are often excluded from clinical trials. Real-world evidence can provide valuable insights into drug persistence, patient-reported outcomes, and the safety profile of ixekizumab in routine clinical practice [13,14,15].

One aspect of treatment that has garnered increasing attention is the role of induction regimens in optimizing therapeutic outcomes with biologics. Induction therapy, typically involving higher or more frequent dosing during the initial treatment phase, aims to achieve rapid disease control and may enhance long-term efficacy and patient adherence [10]. However, the necessity and clinical benefit of the induction phase, particularly in comparison to maintenance dosing alone, have not been comprehensively evaluated in real-world settings.

This study aims to address this gap by comparing the effectiveness and safety of ixekizumab in patients with moderate-to-severe plaque psoriasis who received the treatment with and without the induction phase. By analyzing outcomes such as PASI improvement, treatment persistence, adverse events, and patient-reported satisfaction, this investigation seeks to determine whether induction therapy offers significant advantages over direct maintenance dosing.

The results of this study could have important implications for clinical decision-making and treatment optimization in psoriasis management. If induction therapy is shown to confer substantial benefits, it could reinforce current guidelines and highlight the importance of adherence to recommended dosing schedules. Conversely, if similar outcomes are observed with maintenance dosing alone, this could simplify treatment protocols and reduce the economic burden associated with biologic therapy. By leveraging real-world data, this research contributes to the growing body of evidence needed to tailor psoriasis treatment to individual patient needs and improve long-term outcomes.

## 2. Materials and Methods

This investigation was designed as an observational, descriptive, multicenter study encompassing 183 patients diagnosed with moderate-to-severe plaque psoriasis who underwent treatment with ixekizumab over a five-year period at three tertiary hospitals in Andalucía, Spain.

In this real-world clinical practice study, two groups were established: the first group followed a dosage regimen adjusted according to the drug’s prescribing information, which included an induction phase. In this group, the recommended dose was 160 mg administered subcutaneously (two 80 mg injections) in week 0, followed by 80 mg (one injection) in weeks 2, 4, 6, 8, 10, and 12. This was subsequently followed by a maintenance treatment of 80 mg (one injection) every 4 weeks. The second group, by contrast, did not undergo an induction phase and started directly with a maintenance dose of 80 mg (one injection) every 4 weeks.

A descriptive analysis of the study population was performed, examining variables such as sex, age, body mass index (BMI), baseline PASI score, Physician’s Global Assessment (PGA) score, clinical manifestations, difficult-to-treat locations, presence of psoriatic arthritis, associated comorbidities, and prior treatments. These variables were analyzed in two patient groups stratified by their treatment initiation regimen: with induction therapy as per the standard dosing schedule (date sheet) and without induction.

Categorical variables were described using proportions, while continuous variables were summarized with means and standard deviations. Drug survival was assessed in patients at risk over the five-year period. Treatment continuity rates were analyzed using Kaplan–Meier survival curves, and predictors of drug survival were identified through univariate Cox regression analysis.

Statistical analyses were performed using IBM SPSS Statistics (version 25.0) and GraphPad Prism (version 8.0).

The study was approved by the Ethics Committee of the Hospital Universitario San Cecilio in Granada (approval code: DERM_HUSC_2024) and was conducted in accordance with the principles outlined in the Declaration of Helsinki.

## 3. Results

The study included a total of 183 patients, of whom 156 (85.25%) initiated treatment with induction therapy, while 27 (14.75%) began without induction. Baseline characteristics of patients in both groups are summarized in Table 1.

In both groups, the majority of patients were male, with 64.48% in the induction group and 58.74% in the non-induction group. The mean age in the non-induction group was 54.71 ± 13.23 years, while in the induction group, it was 51.83 ± 13.33 years, with no significant differences observed between the two groups.

Similarly, no statistically significant differences were found in BMI (28.28 ± 6.33 in the induction group and 30.33 ± 5.07 in the non-induction group). However, the main differences were identified in baseline PASI and PGA scores, as shown in Table 1. Most patients in both groups presented with plaque psoriasis.

The scalp was the most challenging site to treat in both groups, followed by the genital area in the induction group and the nails in the non-induction group.

One of the main characteristics of the series presented was the percentage of patients with psoriatic arthritis (50% in both groups), with a predominance of peripheral psoriatic arthritis. Comorbidities associated with metabolic syndrome and cardiovascular risk were prevalent in both groups, as were psychiatric conditions (anxiety, depression, etc.) in the induction group.

Globally, seven patients had a history of hematological or solid organ neoplasms, all of whom were in remission at the time of the study.

Regarding prior treatments, the number and distribution of previous therapies for each patient group are presented in Table 2.

In the induction group, 30.13% were biologic-naïve, while 13.47% of the patients in this group were considered multi-failure patients, having undergone three or more prior lines of biologic therapy. In the group of patients who did not receive induction therapy, only 7.41% were biologic-naïve, whereas the percentage of multi-failure patients was similar to the previous group (14.81%).

Regarding effectiveness, objective parameters such as PASI, BSA, and PGA were measured, along with a patient-reported outcome (PRO): pruritus assessed using a visual analog scale (VAS). All parameters demonstrated parallel evolution in both the group of patients who had undergone induction and the group that had not. The DLQI was documented in only 36 patients, all of whom had received induction therapy. This limited data collection was due to the small sample size of patients with induction and the absence of recorded data in the group without induction.

**Figure 1:**
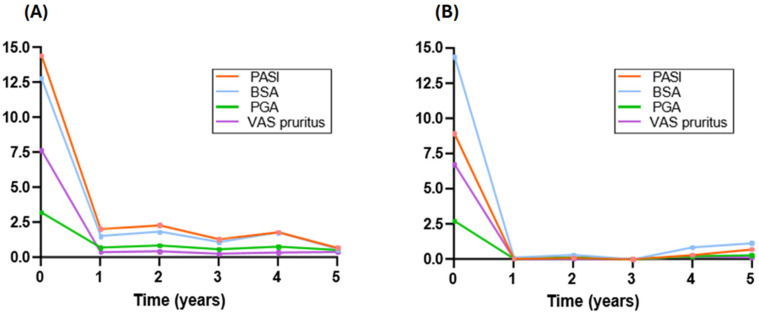
Representation of the parameters PASI, BSA, PGA and VAS pruritus over 5 years in patients with induction (**A**) and in patients without induction (**B**).

The mean baseline PASI in the induction group was 14.42 ± 10.47, while in the non-induction group, it was 8.95 ± 3.96. Interestingly, despite the lower PASI, the BSA was higher in the non-induction group, with a mean of 14.37 ± 9.08. The progression of all parameters is illustrated in Figure 1 and detailed in Table 3 and Table 4, respectively.

**Figure 2:**
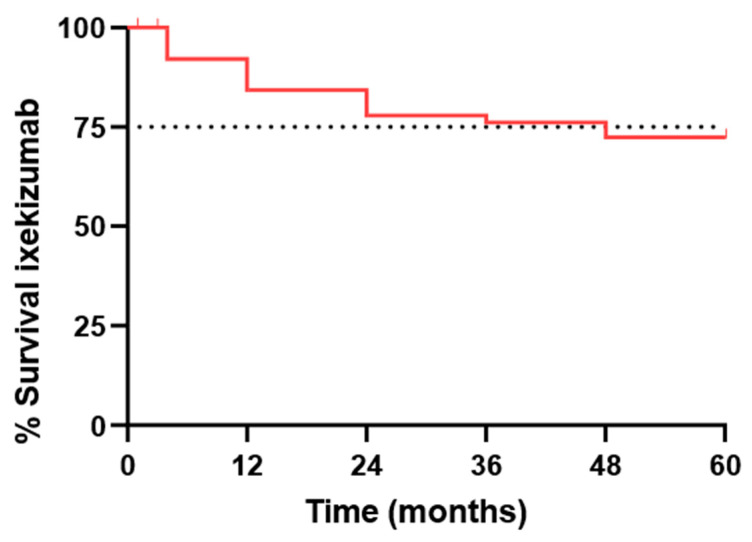
Survival rate of ixekizumab in total patients (n = 183).

For drug survival analysis, data were examined for all patients (n = 183), regardless of the initial dosing regimen. During the study period, 41 patients discontinued treatment with ixekizumab. The discontinuation rates and associated risk percentages are detailed in Table 5 and illustrated in Figure 2.

**Figure 3:**
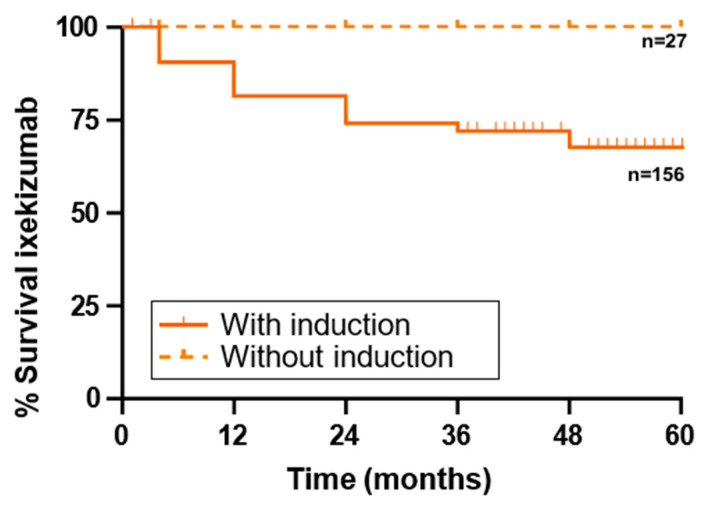
Survival rate of ixekizumab in patients with induction vs. without induction.

A comparative analysis of survival rates between patients who initiated ixekizumab with induction versus those without induction is provided in Table 6 and Figure 3.

Regarding the number of patients who discontinued drug use, there were statistically significant differences between both groups, since all discontinuations belonged to the group of patients who had received induction. In total, 14 patients discontinued treatment due to primary failure and 27 due to secondary failure.

## 4. Discussion

The findings of this study provide valuable insights into the differential outcomes of ixekizumab in patients with moderate-to-severe plaque psoriasis, particularly when comparing induction versus non-induction treatment regimens. Notably, patients who initiated therapy without induction presented with lower baseline PASI and PGA scores but had undergone more previous biologic treatment lines, which may have influenced their treatment responses and clinical trajectories.

### 4.1. Effectiveness of Ixekizumab with and Without Induction Therapy

A systematic review and the LOTIXE study highlight the long-term effectiveness of ixekizumab for moderate-to-severe plaque psoriasis. Ixekizumab, an anti-IL-17A monoclonal antibody, achieved significant responses, with 94.3% of patients attaining PASI75, 85.1% reaching PASI90, and 71.8% achieving PASI100 after 24 months of treatment. Improvements were observed as early as week 4 and were sustained over two years. Notably, patient-reported quality of life, assessed via the DLQI, significantly improved, with effects maintained in the long term [13,15].

These results are consistent with both induction and non-induction patients achieving sustained therapeutic goal over 5 years with the exception of patients who had received induction in the second year in our study.

Both biologic-naïve and biologic-experienced patients demonstrated similar efficacy, and drug retention rates were high at 96%, with minimal discontinuations due to adverse events [13].

Comparative real-world evidence suggests ixekizumab exhibits greater treatment adherence and persistence compared to TNF inhibitors, although data on comparisons with newer IL-23 inhibitors remain limited [15,16,17,18].

Lower baseline PASI and PGA scores in the non-induction group may reflect a less severe initial disease burden, but the higher number of prior biologic failures in this group underscores the complexity of their disease management.

### 4.2. Long-Term Efficacy and Drug Survival

The narrative review by Reich et al. [15] on real-world evidence for ixekizumab in the treatment of psoriasis and psoriatic arthritis (PsA) highlights a substantial body of studies focused on treatment patterns, particularly drug persistence. Their findings suggest that newer IL inhibitors, such as ixekizumab, may exhibit higher persistence compared to TNF inhibitors. However, these conclusions are limited by challenges such as potential misclassification, inconsistent definitions of persistence, and the predominant reliance on U.S.-based claims databases, which restricts the generalizability of the results to broader populations [19,20,21]. Despite these limitations, the findings align with the results from both groups in our study.

Notably, ixekizumab demonstrated comparable or even superior persistence compared to other biologics, along with lower treatment-switching rates. Additionally, it was associated with high adherence and effectiveness, particularly in biologic-naïve patients. The retention rates observed in the non-induction group further suggest that ixekizumab’s long-term benefits may be linked to its initial dosing strategy. This is especially relevant when considering factors such as baseline disease severity, prior treatment failures, and overall disease burden.

Gargiulo et al. [22] analyzed the drug survival of interleukin inhibitors (IL-12/23, IL-17, and IL-23) for moderate-to-severe plaque psoriasis in a real-world, multicenter cohort of 5932 treatment courses. Among anti-IL-17 agents, ixekizumab showed promising results with a 4-year drug survival rate of 82.6%, second only to risankizumab. Stratified analysis revealed ixekizumab had an 87% survival rate when considering only discontinuations due to ineffectiveness. This performance aligns with its high efficacy, as demonstrated in clinical trials and other real-world studies. Ixekizumab was one of the most commonly prescribed biologics in this cohort, largely attributed to its strong therapeutic profile and tolerability. Drug survival, a key indicator of sustained efficacy and patient adherence, was enhanced in bio-naïve patients compared to those switching from other treatments. Overall, the findings emphasize the robust long-term persistence of ixekizumab among IL-17 inhibitors, making it a critical option in the therapeutic landscape of psoriasis.

Although not the aim of this study, as can be seen in Table 1, erythrodermic and pustular forms were more frequent in the induction regimen. Pustular and erythrodermic psoriasis are rare and difficult-to-treat conditions. It has recently been shown that interleukin (IL)-17 inhibitors can be very effective among patients with these forms of psoriasis. Avallone et al. have just published a study where there was a consistent trend towards a higher rate of PASI 100 responses in the patients treated with IL-17 inhibitors compared with those treated with IL-23 inhibitors, and the other efficacy outcomes showed a similar trend. There was no significant between-drug class difference in efficacy at any of the time points in the erythrodermic psoriasis cohort, whereas PASI 90 and PASI 100 response rates were significantly higher among the pustular psoriasis patients receiving IL-17 inhibitors at week 12. In any case, our view is that in these particular forms, since they are off-label, they should be managed as far as possible by induction therapy [23].

### 4.3. Safety Profile of Ixekizumab and Patient’s Reported Outcomes

The safety data reaffirm ixekizumab’s established tolerability, with no unexpected adverse events reported in this cohort. The absence of mucosal candidiasis or inflammatory bowel disease reactivation aligns with prior studies emphasizing the importance of appropriate patient selection to mitigate these risks [15,16,17,18,19].

Patient-reported outcomes (PROs) have been underexplored, despite the importance of addressing PsO’s impact on quality of life. Our series is not an exception and although DLQI and AVS pruritus values are periodically collected, the results are not comparable with many clinical practice series already published. Economic analyses indicated ixekizumab’s direct drug costs as a primary factor in treatment expenses, though its high effectiveness and adherence could mitigate long-term costs.

### 4.4. Study Limitations

Several limitations should be acknowledged.

First, the observational and retrospective design introduces inherent biases, including the potential for selection bias and confounding factors. The non-randomized allocation of patients to induction versus non-induction groups further complicates direct comparisons, as baseline differences in disease severity and treatment history may have influenced outcomes.

Second, the relatively small sample size, particularly in the non-induction group, limits the statistical power of subgroup analyses and reduces the generalizability of the findings.

Finally, the inclusion of only three centers restricts the geographic and demographic diversity of the study population, making it difficult to extrapolate results to broader patient populations.

## 5. Conclusions

This study provides valuable insights into the real-world effectiveness and safety of ixekizumab in moderate-to-severe plaque psoriasis, with particular emphasis on the role of induction therapy. While induction therapy appears to enhance initial PASI responses, meaningful long-term improvements can also be achieved without induction, particularly in patients with lower baseline disease activity. These findings underscore ixekizumab’s versatility and durability as a therapeutic option, even in treatment-experienced populations.

Future research should address the limitations of this study by incorporating larger, more diverse patient cohorts and prospective designs. Additionally, the inclusion of patient-reported outcomes and quality-of-life measures will be critical for capturing the full impact of ixekizumab therapy on patients’ lives. By addressing these gaps, future studies can further refine treatment strategies and optimize outcomes for patients with psoriasis.

## Figures and Tables

**Table 1:** Demographic and clinical characteristics of patients.

		With Induction			Without Induction		
		n	DE	%	n	DE	%
**n**	183	156	-	85.25	27	-	14.75
**Gender**	Male	118	-	64.48	16	-	8.74
	Women	54	-	29.51	11	-	6.01
**Age**		51.83	13.33	-	54.71	13.23	-
**BMI (kg/m^2^)**		28.86	6.33	-	30.33	5.07	-
**Baseline PASI**		14.35	10.59	-	8.95	3.96	-
**Baseline PGA**		3.40	1.10	-	2.74	0.71	-
**Clinical type**	Plaque	149	-	95.51	27	-	100.00
	Pustular	4	-	2.56	0	-	0.00
	Erythroderma	10	-	6.41	2	-	7.41
**Difficult to treat location**	Inverse	20	-	12.82	5	-	18.52
	Genital	29	-	18.59	4	-	14.81
	Scalp	61	-	39.10	27	-	100.00
	Nail	28	-	17.95	13	-	48.15
	Palm-plantar	22	-	14.10	0	-	0.00
	Non PsA	48	-	26.23	10	-	5.46
	Peripheral	44		24.04	8		4.37
**Psoriatic Arthritis** **(PsA)**	Axial	2		1.09	0		0.00
	Mixed	2		1.09	2		1.09
**Comorbidities**	Diabetes	21	-	11.48	3	-	1.64
	Hypertension	47	-	25.68	8	-	4.37
	Dyslipidemia	25	-	13.66	6	-	3.28
	Obesity	37	-	20.22	10	-	5.46
	Metabolic syndrome	16	-	8.74	5	-	2.73
	Nonalcoholic fatty liver disease	6	-	3.28	3	-	1.64
	Neoplasia	4	-	2.19	3	-	1.64
	Psychiatric	31	-	16.94	6	-	3.28
	Hypothyroidism	9	-	4.92	2	-	1.09
	Inflammatory bowel disease	1	-	0.55	0	-	0.00
	Others	34	-	18.58	3	-	1.64

**Table 2:** Number of prior treatments in patients with and without induction.

		With Induction		Without Induction	
		n (Patients)	%	n (Patients)	%
Previous systemic treatments	0	11	7.05	0	0.00
	1	59	37.82	2	7.41
	2	59	37.82	14	51.85
	3	22	14.10	10	37.04
	4	5	3.21	1	3.70
Previous biological treatments	0	47	30.13	2	7.41
	1	59	37.82	13	48.15
	2	29	18.59	8	29.63
	3	15	9.62	4	14.81
	4	6	3.85	0	0.00

**Table 3:** PASI, BSA, PGA and EVA pruritus in patients with induction for 5 years.

	Baseline	1 Year	2 Years	3 Years	4 Years	5 Years
**PASI**	14.42 ± 10.47 (n = 156)	2.03 ± 4.04 (n = 106)	2.30 ± 5.34 (n = 81)	1.31 ± 2.65 (n = 59)	1.81 ± 4.35 (n = 39)	0.68 ± 0.89 (n = 26)
**BSA**	12.79 ± 13.36 (n = 156)	1.49 ± 3.31 (n = 100)	1.81 ± 4.18 (n = 80)	1.06 ± 3.00 (n = 58)	1.75 ± 3.57 (n = 41)	0.56 ± 0.71 (n = 26)
**PGA**	3.23 ± 1.17 (n = 156)	0.70 ± 1.04 (n = 100)	0.84 ± 1.09 (n = 78)	0.57 ± 0.80 (n = 58)	0.76 ± 1.02 (n = 41)	0.52 ± 0.51 (n = 26)
**VAS pruritus**	7.64 ± 1.88 (n = 37)	0.37 ± 0.84 (n = 35)	0.43 ± 0.97 (n = 30)	0.26 ± 0.53 (n = 26)	0.35 ± 0.60 (n = 17)	0.38 ± 0.50 (n = 13)

Data are represented as mean and standard deviation.

**Table 4:** PASI, BSA, PGA and EVA pruritus in patients without induction for 5 years.

	Baseline	1 Year	2 Years	3 Years	4 Years	5 Years
**PASI**	8.95 ± 3.96 (n = 27)	0.04 ± 0.20 (n = 24)	0.11 ± 0.32 (n = 19)	0.0 ± 0.0 (n = 18)	0.31 ± 0.63 (n = 13)	0.71 ± 1.25 (n = 7)
**BSA**	14.37 ± 9.08 (n = 27)	0.13 ± 0.61 (n = 24)	0.32 ± 0.95 (n = 19)	0.0 ± 0.0 (n = 18)	0.85 ± 1.72 (n = 13)	1.14 ± 2.04 (n = 7)
**PGA**	2.74 ± 0.71 (n = 27)	0.04 ± 0.20 (n = 24)	0.16 ± 0.50 (n = 19)	0.0 ± 0.0 (n = 18)	0.23 ± 0.44 (n = 13)	0.29 ± 0.49 (n = 7)
**VAS pruritus**	6.73 ± 2.09 (n = 27)	0.04 ± 0.20 (n = 24)	0.05 ± 0.23 (n = 19)	0.0 ± 0.0 (n = 18)	0.23 ± 0.60 (n = 13)	0.14 ± 0.38 (n = 7)

Data are represented as mean and standard deviation.

**Table 5:** Survival analysis in patients treated with ixekizumab (n = 183).

	N of Patients Who Discontinue	N of Patients at Risk^2^	Survival Rate (95%CI)
**Baseline**	0	**-**	**-**
**4 months**	14	176	92.04 (3.16–5.10)
**12 months**	13	153	84.22 (4.68–6.39)
**24 months**	9	116	77.69 (5.73–7.33)
**36 months**	2	88	75.92 (6.03–7.62)
**48 months**	3	61	72.19 (6.84–8.51)
**60 months**	0	41	-
**Total N**	41 (22.40%)		

**Table 6:** Survival rate of ixekizumab in patients with induction vs. without induction.

*Initial Dose* *Time*		With Induction			Without Induction	
	n = 156 (85.25%)				n = 27 (14.75%)	
	N of Patients Who Discontinued Drug Use	N of Patients at Risk^2^	Survival Rate (95%CI)	N of Patients Who Discontinued Drug Use	N of Patients at Risk^2^	Survival Rate (95%CI)
**Baseline**	0	**-**	**-**	0	**-**	**-**
**4 months**	14	149	90.60 (3.72–5.95)	0	27	100
**12 months**	13	129	81.47 (5.43–7.32)	0	24	100
**24 months**	9	97	73.91 (6.59–8.29)	0	19	100
**36 months**	2	70	71.80 (6.93–8.61)	0	18	100
**48 months**	3	48	67.31 (7.91–9.64)	0	13	100
**60 months**	0	34	-	0	7	100
**Total N**	41			0		
**Log-rank (Mantel–Cox) test**						
Chi square			8.621			
*p* value			0.0033			
**Hazard Ratio (Mantel–Haenszel)**			**With induction vs.** **Without induction**		**With induction vs.** **Without induction**	
Ratio			3.614		0.2767	
CI 95%			1.533 to 8.522		0.1173 to 0.6523	

## Data Availability

The raw data supporting the conclusions of this article will be made available by the authors on request.
